# Association of Cumulative Multimorbidity, Glycemic Control, and Medication Use With Hypoglycemia-Related Emergency Department Visits and Hospitalizations Among Adults With Diabetes

**DOI:** 10.1001/jamanetworkopen.2019.19099

**Published:** 2020-01-10

**Authors:** Rozalina G. McCoy, Kasia J. Lipska, Holly K. Van Houten, Nilay D. Shah

**Affiliations:** 1 Division of Community Internal Medicine, Department of Medicine, Mayo Clinic, Rochester, Minnesota; 2 Division of Health Care Policy and Research, Department of Health Sciences Research, Mayo Clinic, Rochester, Minnesota; 3 Robert D. and Patricia E. Kern Center for the Science of Health Care Delivery, Mayo Clinic, Rochester, Minnesota; 4 Section of Endocrinology, Department of Internal Medicine, Yale School of Medicine, New Haven, Connecticut; 5 OptumLabs, Cambridge, Massachusetts

## Abstract

**Importance:**

Severe hypoglycemia is a serious and potentially preventable complication of diabetes, with some of the most severe episodes requiring emergency department (ED) care or hospitalization. A variety of health conditions increase the risk of hypoglycemia. People with diabetes often have multiple comorbidities, and the association of such multimorbidity with hypoglycemia risk in the context of other risk factors is uncertain.

**Objective:**

To examine the associations of age, cumulative multimorbidity, glycated hemoglobin (HbA_1c_) level, and use of glucose level–lowering medication with hypoglycemia-related ED visits and hospitalizations.

**Design, Setting, and Participants:**

Cohort study of claims and laboratory data from OptumLabs Data Warehouse, an administrative claims database of commercially insured and Medicare Advantage beneficiaries in the United States. Participants were adults (aged ≥18 years) with diabetes who had an available HbA_1c_ level result in 2015. Data from January 1, 2014, to December 31, 2016, were analyzed. Final analyses were conducted from December 2017 to September 2018.

**Main Outcomes and Measures:**

This study calculated rates of hypoglycemia-related ED visits and hospitalizations during the year after the index HbA_1c_ level was obtained, stratified by patient demographic characteristics, diabetes type, comorbidities (from 16 guideline-specified high-risk conditions), index HbA_1c_ level, and glucose level–lowering medication use. The association of each variable with hypoglycemia-related ED and hospital care was examined using multivariable Poisson regression analysis overall and by diabetes type.

**Results:**

The study cohort was composed of 201 705 adults with diabetes (mean [SD] age, 65.8 [12.1] years; 102 668 [50.9%] women; 118 804 [58.9%] white; mean [SD] index HbA_1c_ level, 7.2% [1.5%]). Overall, there were 9.06 (95% CI, 8.64-9.47) hypoglycemia-related ED visits and hospitalizations per 1000 persons per year. The risk of hypoglycemia-related ED visits and hospitalizations was increased by age 75 years or older (incidence rate ratio [IRR], 1.56 [95% CI, 1.23-2.02] vs 18-44 years), black race/ethnicity (IRR, 1.30 [95% CI, 1.16-1.46] vs white race/ethnicity), lower annual household income (IRR, 0.63 [95% CI, 0.53-0.74] for ≥$100 000 vs <$40 000), number of comorbidities (increasing from IRR of 1.66 [95% CI, 1.42-1.95] in the presence of 2 comorbidities to IRR of 4.12 [95% CI, 3.07-5.51] with ≥8 comorbidities compared with ≤1), prior hypoglycemia-related ED visit or hospitalization (IRR, 6.60 [95% CI, 5.77-7.56]), and glucose level–lowering treatment regimen (IRR, 6.73 [95% CI, 4.93-9.22] for sulfonylurea; 12.53 [95% CI, 8.90-17.64] for basal insulin; and 27.65 [95% CI, 20.32-37.63] for basal plus bolus insulin compared with other medications). Independent of these factors, having type 1 diabetes was associated with a 34% increase in the risk of hypoglycemia-related ED visits or hospitalizations (IRR, 1.34 [95% CI, 1.15-1.55]). The index HbA_1c_ level was associated with hypoglycemia-related ED visits and hospitalizations when both low (IRR, 1.45 [95% CI, 1.12-1.87] for HbA_1c_ level ≤5.6% vs 6.5%-6.9%) and high (IRR, 1.24 [95% CI, 1.02-1.50] for HbA_1c_ level ≥10%).

**Conclusions and Relevance:**

In this cohort study of adults with diabetes, the risk of an ED visit or hospitalization for hypoglycemia appeared to be highest among patients with type 1 diabetes, multiple comorbidities, prior severe hypoglycemia, and sulfonylurea and/or insulin use. At-risk patients may benefit from individualized treatment regimens to decrease their risk of hypoglycemia.

## Key Points

**Question:**

Among adults with diabetes, what is the association of multimorbidity with the risk of severe hypoglycemia in the context of other frequently encountered hypoglycemia risk factors, such as age, diabetes type, prior hypoglycemia, glycemic control, and high-risk medication use?

**Findings:**

In this cohort study of 201 705 adults with diabetes in the United States, most individuals had additional chronic health conditions. Cumulative multimorbidity was associated with hypoglycemia-related emergency department visits and hospitalizations independent of other factors.

**Meaning:**

Clinicians may want to consider a broad range of hypoglycemia risk factors, particularly cumulative multimorbidity, when developing a diabetes treatment plan and prioritize prescribing medications with a lower risk for hypoglycemia.

## Teaser

This cohort study examines the associations of age, cumulative multimorbidity, glycated hemoglobin level, and use of glucose level–lowering medication with hypoglycemia-related emergency department visits and hospitalizations among adults with diabetes.

## Introduction

Severe hypoglycemia is associated with poor health outcomes^1,2,3,4,5^ and decreased quality of life^6^ in patients with diabetes. Multiple factors increase the risk of severe hypoglycemia, including older age, a variety of health conditions, social determinants of health, sulfonylurea or insulin therapy, and intensive glycemic control.^7^ Clinical practice guidelines recommend that glycemic targets be relaxed and treatment regimens be deintensified in patients who are older or have health conditions or social situations that increase their risk of hypoglycemia.^8,9,10,11,12,13^ Yet, the contributions of patient-, disease-, and treatment-related factors to severe hypoglycemia are not well defined. Better understanding of the wide range of factors contributing to the risk of experiencing severe hypoglycemia, particularly symptoms severe enough to require emergency department (ED) care or hospitalization, can help inform and enhance clinical decision-making.

The American Diabetes Association (ADA), American Geriatrics Society (AGS), and US Department of Veterans Affairs/Department of Defense (VA/DoD) specify 16 chronic health conditions that warrant consideration of more relaxed glycemic targets and careful selection of glucose level–lowering therapy.^8,9,11,12^ However, there are limited contemporary data about the absolute and relative consequences of these health conditions individually and in the context of multimorbidity on the risk of experiencing hypoglycemia requiring ED or hospital care. Furthermore, using individual comorbidities to risk stratify patients in routine clinical practice may be cumbersome, particularly given the multiple possible permutations of comorbidities. A simpler comorbidity count may be easier to implement in clinical practice, but whether such a comorbidity count is associated with hypoglycemia-related health care use is not known, especially because there is wide variation in the degree of severity and functional decrement that any health condition can incur.

To address these knowledge gaps, we compared the rates of hypoglycemia-related ED visits and hospitalizations experienced by adults with diabetes in the United States as a function of their demographic characteristics (age and race/ethnicity), diabetes type, health status (examining the 16 guideline-specified high-risk conditions separately and as a total count of comorbidities), prior episodes of severe hypoglycemia-related ED visits and hospitalizations, type of glucose level–lowering treatment regimen, and level of glycemic control. Our objectives were as follows: (1) to evaluate the associations of key patient-, disease-, and treatment-related factors with hypoglycemia-related ED visits and hospitalizations in contemporary clinical practice, focusing specifically on comorbidities mentioned in clinical practice guidelines yet not previously examined in parallel and (2) to examine whether comorbidity count alone, irrespective of the specific conditions composing it, is associated with the risk of hypoglycemia-related ED and hospital care. Up to 95% of severe hypoglycemic events do not result in an ED or hospital encounter and are treated outside of the medical system.^14,15,16^ Hypoglycemic events that require ED-level or hospital-level care are likely to be severe, burdensome to patients (by nature of requiring a higher level of care), associated with great cost, and potentially affect the most vulnerable patients (ie, those unable or unsafe to remain at home after an event). These data may help clinicians and health systems better assess the risk of hypoglycemia-related ED visits and hospitalizations and proactively modify treatment regimens to reduce that risk.

## Methods

### Study Design

This retrospective cohort study of medical and pharmacy claims and laboratory results data from OptumLabs Data Warehouse (OLDW) was performed between January 1, 2014, and December 31, 2016. The dates of analysis were December 2017 to September 2018. The index date of cohort entry was set to the last available glycated hemoglobin (HbA_1c_) level result in 2015. Baseline variables were ascertained during the 12 months preceding the index date, and ED visits and hospitalizations were queried during the 12 months after the index date (eFigure 1 in the Supplement). The OLDW includes deidentified claims and laboratory data for commercially insured and Medicare Advantage beneficiaries in a large, private, US health plan.^17^ It contains longitudinal health information on enrollees, representing a diverse mixture of ages, races/ethnicities, and geographic regions across the United States. All study data were accessed only after the data had been deidentified, consistent with a Health Insurance Portability and Accountability Act expert deidentification determination, and when appropriate legal, regulatory, and contractual permissions were met.^18^ As such, this study was exempt from Mayo Clinic Institutional Review Board review and informed consent was not required. We used the Strengthening the Reporting of Observational Studies in Epidemiology (STROBE) guidelines for cohort studies to guide the reporting of this study.

### Study Population

We identified adults (aged ≥18 years) with diabetes who had an available HbA_1c_ level test result between January 1, 2015, and December 31, 2015; if multiple results were available, the last HbA_1c_ level test result of the 2015 calendar year was set as the index date. Participants also had at least 12 months of medical and pharmacy claims data before and after the index HbA_1c_ level test result. The diagnosis of diabetes was established using Healthcare Effectiveness Data and Information Set criteria^19^ applied to 2013-2014 claims. Based on *International Classification of Diseases* (*ICD*) codes, patients with only gestational diabetes (*ICD-9* 648.8x and *ICD-10* O24.4xx) were not included. Diabetes type was ascertained using a modified algorithm adapted from work by Klompas et al^20^ and by Schroeder et al.^21^ Diabetes was categorized as type 1 if most evaluation and management office visit diagnosis codes were for type 1 diabetes during the baseline 12-month period, and the patient had at least 1 insulin claim. In the event of an equal number of type 1 and type 2 diabetes codes, we classified patients as having type 1 diabetes if they used bolus insulin and had no pharmacy fills for sulfonylurea. All others were categorized as having type 2 diabetes.

### Primary Outcome

The primary outcome was the number of ED visits or hospitalizations with the primary or first diagnosis of hypoglycemia during the 12 months after the index HbA_1c_ level result, which was reported per 1000 persons per year. Emergency department visits that resulted in hospitalization were counted as hospitalizations only (ie, were counted once). Events were identified using the algorithm by Ginde et al^22^ for *ICD-9* codes and corresponding *ICD-10* codes (eTable 1 in the Supplement).

### Independent Variables

We examined the presence of the following 16 health conditions specified by the ADA, AGS, and/or US VA/DoD guidelines during the 12 months preceding the index HbA_1c_ level test date: dementia, end-stage renal disease (ESRD), chronic kidney disease (CKD) stages 3 to 4, myocardial infarction, heart failure, cerebrovascular disease, chronic obstructive pulmonary disease, cancer (except for nonmelanoma skin cancer), advanced liver disease (cirrhosis), proliferative retinopathy, peripheral neuropathy, hypertension, arthritis, urinary incontinence, depression, and falls (eTable 1 in the Supplement).^9,11,12^ Because administrative data cannot capture disease severity, end-stage illnesses were conservatively considered together with non–end-stage states except for ESRD. Hypoglycemia-related ED visits and hospitalizations during this period were also identified.

Patient age, sex, race/ethnicity, and annual household income were identified from OLDW enrollment files. The HbA_1c_ level results were categorized as 5.6% or less, 5.7% to 6.4%, 6.5% to 6.9%, 7.0% to 7.9%, 8.0% to 8.9%, 9.0% to 9.9%, and 10.0% or higher.

Diabetes medications were identified from pharmacy fill data during 100 days before the index HbA_1c_ level result. They were classified as sulfonylurea, insulin (basal and/or bolus), or other medications (metformin, dipeptidyl peptidase 4 [DPP-4] inhibitors, glucagon-like peptide 1 [GLP-1] receptor agonists, sodium-glucose cotransporter 2 [SGLT2] inhibitors, α-glucosidase inhibitors, thiazolidinediones, meglitinides, and amylin analogues).

### Statistical Analysis

We calculated overall frequencies (percentages) and means (SDs) for patient characteristics, including age, sex, race/ethnicity, annual household income, comorbidities, history of hypoglycemia-related ED visit or hospitalization, index HbA_1c_ level, and use of glucose level–lowering medication. Crude (unadjusted) rates of hypoglycemia-related ED visits and hospitalizations were calculated and are presented as the total number of events per 1000 persons per year overall and for each characteristic.

Multivariable Poisson regression analysis was used to assess the adjusted associations of patient age, comorbidity profile (evaluated 2 different ways, as described below), number of hypoglycemia-related ED visits and hospitalizations, index HbA_1c_ level, and glucose level–lowering treatment regimen, adjusting for each other and patient sex, race/ethnicity, annual household income, diabetes type, and history of hypoglycemia-related ED visit or hospitalization. The primary analysis evaluated multimorbidity as the total count of health conditions. Results are reported as incidence rate ratios (IRRs) and 95% CIs, with reference groups set to ages 18 to 44 years, male sex, white race/ethnicity, annual household income less than $40 000, 1 or 0 specified comorbidities, no hypoglycemia-related ED visit or hospitalization in the prior year, HbA_1c_ level of 6.5% to 6.9%, and treatment with other glucose level–lowering medication (neither sulfonylurea nor insulin).

In a secondary analysis, each individual health condition was considered separately (reference is absent comorbidity for each) rather than as a total count of comorbidities. To assess for possible interaction between age and the number of comorbidities, multivariable Poisson regression models were run separately for each age group, with comorbidity burden evaluated as a continuous count of comorbidities. All analyses were performed for the overall population (main analysis) and stratified by diabetes type. Analyses were conducted using software programs, including SAS, version 9.4, (SAS Institute Inc) and Stata, version 15.1 (StataCorp LP). All tests were 2 tailed, and *P* < .05 was considered statistically significant.

## Results

### Study Population

The study cohort was composed of 201 705 adults with diabetes (eFigure 2 in the Supplement) (mean [SD] age, 65.8 [12.1] years; 102 668 [50.9%] women; 118 804 [58.9%] white; 7548 [3.7%] with type 1 diabetes) (Table). The mean (SD) index HbA_1c_ level was 7.2% (1.5%). Patients had a mean (SD) of 2.1 (1.5) of the 16 examined guideline-specified high-risk conditions in addition to diabetes; only 9.1% (n = 18 387) had none, and 7.7% (n = 15 598) had 5 conditions or more.

**Table.** Study Population Characteristics and Crude Annual Rates of Hypoglycemia-Related ED Visits and Hospitalizations^a^

Variable	No. (%)	Hypoglycemia-Related ED Visits and Hospitalizations/1000 Persons/y (95% CI)
Overall population	201 705	9.06 (8.64-9.47)
Age, mean (SD), y	65.8 (12.1)	NA
Age group, y		
18-44	11 567 (5.7)	7.00 (5.48-8.53)
45-64	65 993 (32.7)	6.83 (6.20-7.46)
65-74	76 075 (37.7)	9.12 (8.44-9.80)
≥75	48 070 (23.8)	12.50 (11.50-13.50)
Sex		
Female	102 757 (50.9)	9.61 (9.01-10.20)
Male	98 948 (49.1)	8.49 (7.92-9.06)
Race/ethnicity		
White	118 857 (58.9)	8.77 (8.23-9.30)
Black	32 947 (16.3)	13.72 (12.45-14.98)
Hispanic	29 775 (14.8)	6.82 (5.88-7.76)
Asian	11 484 (5.7)	5.49 (4.13-6.84)
Other or unknown	8642 (4.3)	7.7.75 (5.90-9.61)
Annual household income, $		
<40 000	60 838 (30.2)	12.29 (11.41-13.18)
40 000-49 999	19 434 (9.6)	10.60 (9.15-12.05)
50 000-59 999	17 393 (8.6)	9.31 (7.88-10.75)
60 000-74 999	21 296 (10.6)	8.55 (7.30-9.79)
75 000-99 999	26 469 (13.1)	6.99 (5.98-8.00)
≥100 000	42 412 (21.0)	4.53 (3.89-5.17)
Unknown	13 863 (6.9)	10.96 (9.22-12.71)
Diabetes type		
Type 1	7548 (3.7)	32.06 (28.02-36.10)
Type 2	194 157 (96.3)	8.16 (7.76-8.57)
No. of comorbidities, mean (SD)	2.1 (1.5)	NA
No. of comorbidities		
≤1	82 245 (40.8)	3.47 (3.06-3.87)
2	52 941 (26.2)	6.99 (6.28-7.70)
3	33 104 (16.4)	11.66 (10.50-12.82)
4	17 817 (8.8)	17.17 (15.25-19.10)
5	8701 (4.3)	24.71 (21.41-28.01)
6	4056 (2.0)	33.78 (28.12-39.43)
7	1813 (0.9)	38.06 (29.08-47.04)
≥8	1028 (0.5)	57.39 (42.75-72.04)
Comorbidities		
None	18 387 (9.1)	2.66 (1.92-3.41)
Dementia	5638 (2.8)	23.94 (19.91-27.98)
End-stage renal disease	2953 (1.5)	43.35 (35.84-50.86)
Chronic kidney disease stages 3 to 4	22 889 (11.3)	23.07 (21.10-25.04)
Myocardial infarction	7636 (3.8)	24.88 (21.34-28.42)
Heart failure	19 268 (9.6)	22.42 (20.31-24.53)
Cerebrovascular disease, stroke/TIA	24 034 (11.9)	17.81 (16.12-19.50)
Chronic obstructive pulmonary disease	27 012 (13.4)	15.51 (14.03-17.00)
Cancer, except nonmelanoma skin cancer	18 940 (9.4)	9.82 (8.41-11.23)
Cirrhosis	1832 (0.9)	20.20 (13.69-26.70)
Proliferative retinopathy	4446 (2.2)	29.01 (24.01-34.02)
Peripheral neuropathy	48 012 (23.8)	16.97 (15.81-18.14)
Hypertension	168 409 (83.5)	9.93 (9.46-10.41)
Arthritis	43 169 (21.4)	11.86 (10.83-12.89)
Urinary incontinence	7284 (3.6)	14.96 (12.15-17.77)
Depression	21 392 (10.6)	16.31 (14.60-18.03)
Falls	6624 (3.3)	25.66 (21.81-29.52)
Prior severe hypoglycemia–related ED visit or hospitalization within 12 mo	1612 (0.8)	171.22 (151.02-191.42)
Index HbA_1c_ level, mean (SD), %	7.2 (1.5)	NA
Index HbA_1c_ level, %		
≤5.6	10 113 (5.0)	7.91 (6.18-9.64)
5.7-6.4	57 910 (28.7)	5.08 (4.50-5.66)
6.5-6.9	40 557 (20.1)	6.07 (5.31-6.82)
7.0-7.9	48 234 (23.9)	9.58 (8.70-10.45)
8.0-8.9	21 705 (10.8)	15.94 (14.26-17.62)
9.0-9.9	10 553 (5.2)	17.25 (14.74-19.75)
≥10.0	12 633 (6.3)	17.18 (14.89-19.46)
Glucose level–lowering treatment regimen		
No pharmacy fills for glucose level–lowering drugs	38 464 (19.1)	4.11 (3.47-4.75)
≥1 Glucose level–lowering drug filled	163 241 (80.9)	10.22 (9.73-10.71)
No. of glucose level–lowering medication classes, mean (SD)^b^	2.5 (1.7)	NA
Glucose level–lowering medication used^b^		
Sulfonylurea^c^	49 200 (24.4)	5.75 (5.08-6.42)
Basal insulin^c^	10 088 (5.0)	12.39 (10.22-14.56)
Basal insulin plus sulfonylurea^c^	9465 (4.7)	13.42 (11.08-15.75)
Bolus insulin^c^	3134 (1.6)	21.06 (15.98-26.14)
Bolus insulin plus sulfonylurea^c^	590 (0.3)	25.42 (12.56-38.29)
Basal plus bolus insulin^c^	22 238 (11.0)	36.11 (33.61-38.61)
Basal plus bolus insulin plus sulfonylurea^c^	6609 (3.3)	30.87 (26.63-35.10)
Other medications	61 917 (30.7)	0.74 (0.53-0.96)

Abbreviations: ED, emergency department; HbA_1c_, hemoglobin A_1c_; NA, not applicable; TIA, transient ischemic attack.

SI conversion factor: To convert HbA_1c_ to proportion of total hemoglobin multiply by 0.01.

^a^ Listed are crude (unadjusted) rates of hypoglycemia-related ED visits and hospitalizations and the proportion of patients with any hypoglycemia-related visit during 12 months.

^b^ Distribution of glucose level–lowering treatment regimens was calculated for patients with pharmacy fills only.

^c^ Patients may also be receiving additional medications other than sulfonylurea or insulin.

### Crude Rates of Hypoglycemia-Related ED Visits and Hospitalizations

**Figure 1.**
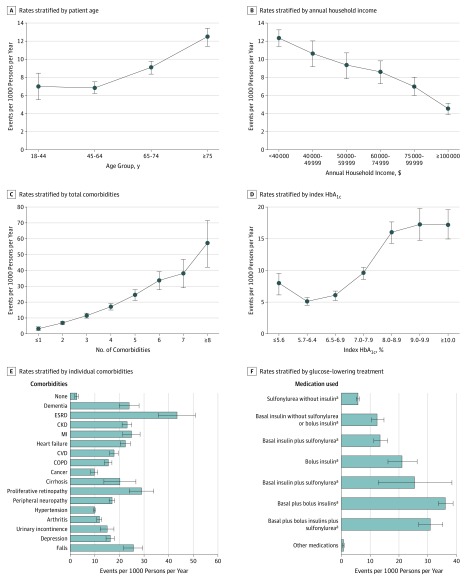
Crude Rates of Hypoglycemia-Related Emergency Department Visits and Hospitalizations Rates of hypoglycemia-related emergency department visits and hospitalizations were calculated as the total number of emergency department visits and hospitalizations with the primary (first) diagnosis of hypoglycemia per 1000 persons with the characteristic of interest (eg, age ≥75 years) per year. CKD indicates chronic kidney disease (stages 3-4); COPD, chronic obstructive pulmonary disease; CVD, cardiovascular disease (stroke/transient ischemic attack); ESRD, end-stage renal disease; HbA_1c_, hemoglobin A_1c_; MI, myocardial infarction; and error bars, 95% CI. ^a^Patients may also be receiving additional medications other than sulfonylurea or insulin.

Overall, the rate of hypoglycemia-related ED visits and hospitalizations was 9.06 (95% CI, 8.64-9.47) per 1000 persons per year, with marked variability as a function of demographic, clinical, and diabetes management characteristics (Table). Crude rates of hypoglycemia-related ED visits and hospitalizations increased with older age (Figure 1A), lower annual household income (Figure 1B), and increasing number of comorbidities (Figure 1C). The index HbA_1c_ level had a U-shaped association with hypoglycemic event rates, with a nadir at an HbA_1c_ level of 5.7% to 6.4% (Figure 1D). Black patients had the highest frequency of hypoglycemia-related ED visits and hospitalizations of all racial/ethnic groups (13.72 [95% CI, 12.45-14.98] per 1000 persons per year compared with 8.77 [95% CI, 8.23-9.30] among white patients) (Table). Patients with type 1 diabetes had more frequent hypoglycemia-related ED visits and hospitalizations than did patients with type 2 diabetes (32.06 [95% CI, 28.02-36.10] vs 8.16 [95% CI, 7.76-8.57] per 1000 persons per year, respectively).

Patients with any of the 16 examined chronic conditions had higher crude rates of hypoglycemia-related ED visits and hospitalizations than patients with none (Figure 1E). Hypoglycemia-related ED visit and hospitalization rates were highest among patients with ESRD (43.35 [95% CI, 35.84-50.86] per 1000 persons per year), proliferative retinopathy (29.01 [95% CI, 24.01-34.02] per 1000 persons per year), falls (25.66 [95% CI, 21.81-29.52] per 1000 persons per year), myocardial infarction (24.88 [95% CI, 21.34-28.42] per 1000 persons per year), dementia (23.94 [95% CI, 19.91-27.98] per 1000 persons per year), and CKD stages 3 to 4 (23.07 [95% CI, 21.10-25.04] per 1000 persons per year) (Table). Patients who had an ED visit or hospitalization for hypoglycemia in the prior year experienced recurrent events most often (171.22 [95% CI, 151.02-191.42] per 1000 persons per year). The rates of hypoglycemia-related ED visits and hospitalizations were lowest among patients treated with medications other than sulfonylurea or insulin (0.74 [95% CI, 0.53-0.96] per 1000 persons per year) and increased with the addition of sulfonylurea, basal insulin, and basal plus bolus insulin regimens to 5.75 (95% CI, 5.08-6.42), 12.39 (95% CI, 10.22-14.56), and 36.11 (95% CI, 33.61-38.61) per 1000 persons per year, respectively (Table and Figure 1F).

Among patients with type 1 diabetes (eFigure 3 in the Supplement), crude rates of hypoglycemia-related ED visits and hospitalizations were highest among those 75 years or older (43.95 [95% CI, 31.11-56.79] per 1000 persons per year) and lowest among those aged 18 to 44 years (17.63 [95% CI, 11.70-23.55] per 1000 persons per year). Crude rates of hypoglycemia-related ED visits and hospitalizations increased with decreasing annual household income and higher number of comorbidities and had a U-shaped association with index HbA_1c_ level (eFigure 3 in the Supplement). Rates among patients with type 2 diabetes were almost identical to those of the total cohort (eFigure 4 in the Supplement).

### Independent Risk Factors for Hypoglycemia-Related ED Visits and Hospitalizations

**Figure 2.**
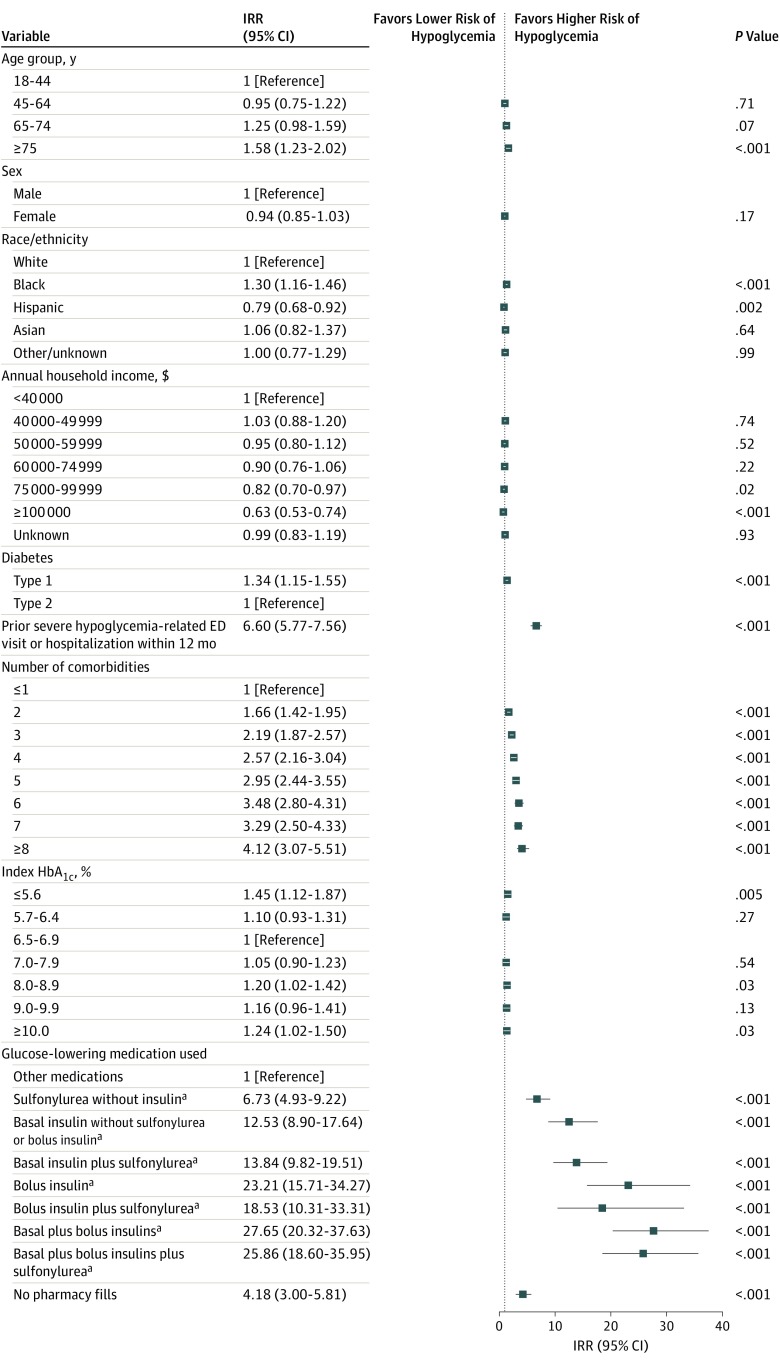
Independent (Adjusted) Patient Risk Factors for Hypoglycemia-Related Emergency Department (ED) Visits and Hospitalizations Shown are adjusted incidence rate ratios (IRRs) (95% CIs) of multivariable Poisson regression analysis examining the association of patient characteristics and potential hypoglycemia risk factors with hypoglycemia-related emergency department visits and hospitalizations, with all factors adjusted for simultaneously. The total number of comorbidities was calculated among dementia, end-stage renal disease, chronic kidney disease stages 3 to 4, myocardial infarction, heart failure, cerebrovascular disease (stroke/transient ischemic attack), chronic obstructive pulmonary disease, cancer (except nonmelanoma skin cancer), cirrhosis, proliferative retinopathy, peripheral neuropathy, hypertension, arthritis, urinary incontinence, depression, and falls. Prior severe hypoglycemia-related ED visit or hospitalization within 12 months was considered separately and thus was not included in the total count of guideline-specified chronic conditions. HbA_1c_ indicates hemoglobin A_1c_. ^a^Patients may also be receiving additional medications other than sulfonylurea or insulin.

Because crude rates of hypoglycemia-related ED visits and hospitalizations may be driven by confounding from concurrent but not independently statistically significant risk factors, we examined the adjusted associations of the aforementioned demographic and clinical characteristics with hypoglycemia-related ED visits and hospitalizations (Figure 2). The association of patient age, annual household income, and index HbA_1c_ level with the risk of hypoglycemia-related ED visits and hospitalizations was attenuated in the adjusted analyses. As shown in Figure 2, age remained a risk factor only for age 75 years or older (IRR, 1.58 [95% CI, 1.23-2.02] vs 18-44 years) but not for younger age groups. The index HbA_1c_ level had a weaker association with hypoglycemia-related ED visits and hospitalizations than other factors, being only significant at HbA_1c_ levels of 5.6% or less and 10.0% or greater (IRRs, 1.45 [95% CI, 1.12-1.87] and 1.24 [95% CI, 1.02-1.50], respectively). Only annual household income of at least $100 000 remained independently associated with hypoglycemia-related ED visits and hospitalizations compared with annual household income of less than $40 000 (IRR, 0.63 [95% CI, 0.53-0.74]).

History of hypoglycemia-related ED visit or hospitalization was associated with increased risk of a recurrent event by an IRR of 6.60 (95% CI, 5.77-7.56) (Figure 2). Other factors associated with increased hypoglycemia-related ED and hospital care risk were black race/ethnicity (IRR, 1.30 [95% CI, 1.16-1.46] vs white race/ethnicity) and type 1 diabetes (IRR, 1.34 [95% CI, 1.15-1.55] vs type 2 diabetes). Hispanic race/ethnicity was associated with lower risk of hypoglycemia-related ED visits and hospitalizations (IRR, 0.79 [95% CI, 0.68-0.92]).

There were important differences in the associations of these variables with the risk of hypoglycemia-related ED visits and hospitalizations between patients with type 1 diabetes and type 2 diabetes (eTable 2 in the Supplement). Older age (specifically, ≥75 years) was associated with increased risk of hypoglycemia-related ED visits and hospitalizations among patients with type 2 diabetes but not type 1 diabetes. Black patients and low-income patients with type 2 diabetes but not type 1 diabetes had a higher risk of hypoglycemia-related ED and hospital care.

The risk of experiencing a hypoglycemia-related ED visit or hospitalization increased progressively with more comorbidities, irrespective of the specific conditions present, overall and stratified by diabetes type (Figure 2 and eTable 2 in the Supplement). Specifically, the risk of hypoglycemia-related ED visit or hospitalization among patients with 2 of the 16 comorbidities was 66% higher (IRR, 1.66 [95% CI, 1.42-1.95]) than among patients with 1 or no comorbidity and increased progressively to 412% higher (IRR, 4.12 [95% CI, 3.07-5.51]) among patients with 8 or more comorbidities. However, there was interaction between patient age and the cumulative comorbidity burden. Although increasing multimorbidity was statistically significantly associated with elevated risk of hypoglycemia-related ED visits and hospitalization at each age level, the increase in risk per each additional comorbidity was progressively higher among younger patients overall and stratified by diabetes type (eTable 3 in the Supplement). Specifically, for each additional comorbidity, the risk of hypoglycemia requiring ED or hospital care increased by 56% among patients aged 18 to 44 years (IRR, 1.55 [95% CI, 1.37-1.77]), 28% among patients aged 45 to 64 years (IRR, 1.28 [95% CI, 1.22-1.34]), 25% among patients aged 65 to 74 years (IRR, 1.25 [95% CI, 1.20-1.30]), and just 8% among patients 75 years or older (IRR, 1.08 [95% CI, 1.03-1.13]).

When we examined the adjusted association of each comorbidity with the risk of hypoglycemia requiring ED visits and hospitalizations (eTable 4 in the Supplement), we found the strongest associations with ESRD, CKD stages 3 to 4, myocardial infarction, and falls; each of these factors was associated with an approximately 40% increase in the risk of hypoglycemia-related ED visits and hospitalizations after adjustment for each other and other comorbidities. Cerebrovascular disease (stroke or transient ischemic attack), chronic obstructive pulmonary disease, peripheral neuropathy, and depression were each associated with a 15% to 30% increase in the risk of hypoglycemia-related ED visits and hospitalization. Dementia, heart failure, cancer, advanced liver disease (cirrhosis), proliferative retinopathy, hypertension, arthritis, and urinary incontinence were not independently associated with the risk of hypoglycemia-related ED visits and hospitalizations. Associations of individual comorbidities with hypoglycemia-related ED visits and hospitalizations for patients with type 1 diabetes and type 2 diabetes separately are listed in eTable 5 in the Supplement.

The most informative factor associated with hypoglycemia-related ED and hospital care was the modality of diabetes management (Figure 2 and eTable 2 and eTable 4 in the Supplement). Specifically, compared with patients treated with medications other than sulfonylurea and insulin, the risk of a hypoglycemia-related ED visit or hospitalization was 6.7-fold higher (IRR, 6.73 [95% CI, 4.93-9.22]) among patients treated with sulfonylurea (without insulin), 12.5-fold higher (IRR, 12.53 [95% CI, 8.90-17.64]) among patients treated with basal insulin (without sulfonylurea or bolus insulin), 13.8-fold higher (IRR, 13.84 [95% CI, 9.82-19.51]) among patients treated with basal insulin plus sulfonylurea, 23.2-fold higher (IRR, 23.21 [95% CI, 15.71-34.27]) among patients treated with bolus insulin, and 27.7-fold higher (IRR, 27.65 [95% CI, 20.32-37.63]) among patients treated with basal plus bolus insulin. Patients who had no pharmacy fills for diabetes medications in the 100 days preceding their index HbA_1c_ level test result had a 4-fold higher (IRR, 4.18 [95% CI, 3.00-5.81]) risk of hypoglycemia-related ED visits and hospitalizations compared with patients who had pharmacy fills for other medications. In a sensitivity analysis that queried medications filled during the 12 months preceding the index HbA_1c_ level test rather than 100 days, thereby probing for medication use with poor adherence, results were unchanged.

## Discussion

In this contemporary US cohort of 201 705 adults with diabetes, the overall crude rate of hypoglycemia requiring ED care or hospitalization was 9.1 per 1000 persons per year, with wide variation as a function of comorbidity burden and the glucose level–lowering treatment regimen. Building on prior work regarding the epidemiology of hypoglycemia,^7,23,24,25,26^ we found that cumulative multimorbidity itself, independent of the specific health conditions composing it, was strongly associated with hypoglycemia-related ED visits and hospitalizations. Additional factors associated with the risk of hypoglycemia-related ED and hospital care were age 75 years or older (particularly among patients with type 2 diabetes), black race/ethnicity (particularly among patients with type 2 diabetes), type 1 diabetes, and prior severe hypoglycemia. However, the strongest association with hypoglycemia-related ED visits and hospitalization was the choice of glucose level–lowering medication (for patients with type 2 diabetes), with use of prandial insulin and, to a lesser degree, basal insulin or sulfonylurea, conferring the highest risk. In contrast, the association of the HbA_1c_ level with hypoglycemia-related ED visits and hospitalizations was weak, particularly among patients with type 2 diabetes, reinforcing the importance of focusing on and modifying treatment regimens (not HbA_1c_ level targets) in patients at risk for hypoglycemia.

### Patient Factors Associated With Hypoglycemia-Related ED Visits and Hospitalizations

Crude numbers of hypoglycemia-related ED visits and hospitalizations increased consistently after age 65 years, but the independent association with age was apparent only for patients 75 years or older compared with patients aged 18 to 44 years. The attenuation of hypoglycemia risk by adjusting for other factors reinforces the multifaceted risk phenotype of older adults, whereby underlying clinical complexity and treatment modality may be more influential than age per se. This is consistent with AGS^11^ and VA/DoD^9^ guidelines, which do not use chronological age as a sole indication for modified treatment targets or regimens but rather focus on comorbidity burden, frailty, and life expectancy.

Consistent with prior studies, we found that the risk of hypoglycemia-related ED visits and hospitalizations was statistically significantly higher among black patients than among other racial/ethnic groups,^25,27,28^ although this finding may also reflect unmeasured differences in health care access and other social determinants of health that cannot be captured by claims data. Patients with type 1 diabetes had 34% higher risk of experiencing a hypoglycemia-related ED visit or hospitalization even after all other factors were accounted for (including glucose level–lowering treatment regimen). These nonmodifiable patient characteristics may serve as warning signs for increased susceptibility to hypoglycemia requiring ED and hospital care and may prompt clinicians to consider potential mitigation strategies, including closer monitoring and more intensive diabetes self-management education and support.

### Multimorbidity and Hypoglycemia-Related ED Visits and Hospitalizations

Most adults with diabetes have multiple chronic conditions in addition to diabetes.^29,30,31^ In our study population, only 9% did not have any of the 16 comorbidities highlighted by the ADA, AGS, and VA/DoD guidelines.^8,9,11,12^ Although not all of the examined health conditions were equally associated with the risk of hypoglycemia-related ED visits and hospitalizations, the cumulative count of comorbidities was independently associated with experiencing a hypoglycemia-related event. Prior studies and clinical guidelines have varied in the specific comorbidities that were considered to reflect heightened hypoglycemia risk, and we believed that our study both quantified the specific associations of these health conditions with hypoglycemia risk and demonstrated that at the population level the total comorbidity burden (count) may be an effective and simple way of identifying highest-risk individuals in need of closer monitoring and potential intervention. In addition, we found that increasing comorbidity had a stronger association with hypoglycemia-related ED and hospital care in younger adults than in older adults. For each additional comorbidity, the risk of hypoglycemia requiring ED and hospital care increased by 56% among patients aged 18 to 44 years, by 28% among patients aged 45 to 64 years, by 25% among patients aged 65 to 74 years, and by just 8% among patients 75 years or older. For us, this finding reinforces our understanding of the degree to which multimorbidity alters the health and health outcomes of younger adults, in whom multimorbidity may reflect worse functional status and greater debility. In addition, younger patients may experience greater burden from multimorbidity if they need to balance the demands of their illness with the activities of everyday life (eg, employment, taking care of family).

### Use of Glucose Level–Lowering Medication and Hypoglycemia-Related ED Visits and Hospitalizations

We built on existing literature identifying sulfonylurea and insulin use as important hypoglycemia risk factors^23,24,25,32^ by examining these medications in parallel with other relevant risk factors, differentiating between basal insulin and basal plus bolus insulin regimens and exploring the implications of concurrent sulfonylurea and insulin use. In both unadjusted and adjusted analyses, patients treated with sulfonylurea, basal insulin, and basal plus bolus insulin regimens experienced incrementally increasing rates of hypoglycemia-related ED visits and hospitalizations. Specifically, in multivariable analysis, sulfonylurea use was associated with an almost 7-fold higher risk of a hypoglycemia-related visit or hospitalization, basal insulin use was associated with a 12.5-fold increase in risk, and basal plus bolus insulin use was associated with an almost 28-fold increase in risk. This increase in hypoglycemia risk is higher than previously described,^23,24,25,32^ but those studies did not directly compare sulfonylurea, basal insulin, multiple daily insulin injections, and other glucose level–lowering drugs with each other and within the same diverse patient population in a real-world setting. We found that for patients with type 2 diabetes, it seems that the addition of sulfonylurea to insulin (whether basal only or basal plus bolus) did not increase the risk of hypoglycemia-related ED and hospital care beyond what was observed for insulin alone.

### HbA_1c_ Level and Hypoglycemia-Related ED Visits and Hospitalizations

We did not find a strong association of the HbA_1c_ level with the risk of hypoglycemia-related ED visits and hospitalizations. This is not surprising because the HbA_1c_ level is a measure of mean blood glucose levels over the preceding 3 months and does not reflect glycemic variability. Furthermore, HbA_1c_ levels may not reliably reflect even average glycemia in patients with multimorbidity,^33^ particularly those with anemia or uremia.^34^ Therefore, rather than focusing on changing HbA_1c_ level targets, clinicians need to assess broad risk factors for hypoglycemia to lower risk. Individualized strategies to reduce hypoglycemia risk may include treatment simplification and/or deintensification,^35,36^ use of noninsulin therapies (when feasible and appropriate), or provision of additional support for monitoring and medication management.^7^ Furthermore, because hypoglycemia-related ED visits and hospitalizations occurred most often among patients with high (not low) HbA_1c_ levels, it is important to screen at-risk patients for hypoglycemia even when their HbA_1c_ level is elevated.

### Limitations

This study has limitations. Like other studies that rely on claims or electronic health record data, our study does not capture the vast majority of severe hypoglycemic events because most do not culminate in an ED visit or hospitalization.^14,15,16^ We also could not identify fatal hypoglycemic events if they did not result in an ED visit or hospitalization. Although events requiring ED or hospital care are by definition severe and have a strong harm on patients, the health care system, and society, these events also do not necessarily reflect the magnitude of hypoglycemia because factors other than blood glucose levels may alter the likelihood of needing ED or hospital care. These factors include patient frailty, clinical complexity, and availability of support. Therefore, associations observed in our study between such factors and hypoglycemia-related ED visits and hospitalizations may not exclusively be due to their potential causal effect on hypoglycemia, but rather on the decision to seek or need ED or hospital care.

Not all disease ascertainment algorithms used in this study were previously validated, nor could they be independently validated through medical record review herein because OLDW is a deidentified administrative claims database. This includes ascertainment of diabetes type, although we adapted previously validated algorithms^20,21^ to be more specific for type 1 diabetes by including not only diagnosis codes but also medications (eg, insulin use). Medication capture may not be complete, as evidenced by the occurrence of hypoglycemia-related ED visits and hospitalizations among patients with no pharmacy fills for glucose level–lowering medications. These patients may obtain medications without a health insurance transaction or claim (ie, purchased through low-cost generic drug programs^37^ or obtained as samples). We also did not consider insulin delivery modality (pens vs vials) or type (human vs analogue insulin), which was beyond the scope of this study. Claims data may not completely reflect a patient’s comorbidity burden because certain conditions (eg, dementia, urinary incontinence, and falls) are often not documented, whereas others may be coded even if precise clinical diagnostic criteria are not met. Glucose levels, which are informative in this context because they provide a more accurate assessment of glycemic control,^33^ were also not available. Nonetheless, this information is readily available at the point of care and as part of population health management, and we think it can support treatment deintensification and other efforts to reduce the burden of hypoglycemia-related ED visits and hospitalizations.

## Conclusions

Cumulative multimorbidity appears to be a strong independent risk factor for hypoglycemia-related ED visits and hospitalizations, particularly among younger adults with diabetes. The risk of hypoglycemia-related ED and hospital care is further increased among patients who are 75 years or older, black, with type 1 diabetes, and with prior severe hypoglycemic events. Yet, we believe that the strongest independent risk factor for hypoglycemia-related ED and hospital care was the choice of glucose level–lowering medication, with highest risk among patients treated with basal plus bolus insulin regimens, followed by basal insulin and sulfonylurea. This finding appears to signal an opportunity for practice improvement. Clinicians caring for patients at risk for hypoglycemia may want to preferentially prescribe medications other than insulin and sulfonylurea whenever possible. Patients who require insulin therapy, including those with type 1 diabetes or ESRD, should be closely monitored and optimally protected against hypoglycemia through careful dosing, diabetes self-management education, hypoglycemia awareness training, and screening for management of social determinants of health that alter hypoglycemia risk.^7^ Hypoglycemia is a common, serious, and potentially preventable complication of diabetes, and we believe that clinicians need to consider a broad range of hypoglycemia risk factors, including multimorbidity, when developing a diabetes treatment plan that optimizes glycemic control while ensuring low risk of adverse events, including hypoglycemia.

## Notes

Supplement. **eTable 1.** ICD-9 and ICD-10 Codes Used to Ascertain Study Conditions **eTable 2.** Adjusted Risk Factors for Hypoglycemia-Related ED Visits/Hospitalizations—Type 1 vs Type 2 Diabetes **eTable 3.** Differential Effect of Multimorbidity as a Function of Age **eTable 4.** Adjusted Risk Factors for Hypoglycemia-Related ED Visits/Hospitalizations—Secondary Analysis Examining Individual Comorbidities in the Overall Population **eTable 5.** Adjusted Risk Factors for Hypoglycemia-Related ED Visits/Hospitalizations—Secondary Analysis Examining Individual Comorbidities Among Patients With Type 1 and Type 2 Diabetes **eFigure 1.** Study Design **eFigure 2.** Study Population **eFigure 3.** Crude Rates of Hypoglycemia-Related ED Visits/Hospitalizations Among Patients With Type 1 Diabetes **eFigure 4.** Crude Rates of Hypoglycemia-Related ED Visits/Hospitalizations Among Patients With Type 2 Diabetes **eReferences.**
