# Supplementary material for: Association of Cumulative Multimorbidity, Glycemic Control, and Medication Use With Hypoglycemia-Related Emergency Department Visits and Hospitalizations Among Adults With Diabetes
Source: JAMA Netw Open. 2020 Jan 10;3(1):e1919099. doi: 10.1001/jamanetworkopen.2019.19099 (PMC6991264; doi:10.1001/jamanetworkopen.2019.19099)
Supplement: Supplement. — eTable 1. ICD-9 and ICD-10 Codes Used to Ascertain Study Conditions eTable 2. Adjusted Risk Factors for Hypoglycemia-Related ED Visits/Hospitalizations—Type 1 vs Type 2 Diabetes eTable 3. Differential Effect of Multimorbidity as a Function of Age eTable 4. Adjusted Risk Factors for Hypoglycemia-Related ED Visits/Hospitalizations—Secondary Analysis Examining Individual Comorbidities in the Overall Population eTable 5. Adjusted Risk Factors for Hypoglycemia-Related ED Visits/Hospitalizations—Secondary Analysis Examining Individual Comorbidities Among Patients With Type 1 and Type 2 Diabetes eFigure 1. Study Design eFigure 2. Study Population eFigure 3. Crude Rates of Hypoglycemia-Related ED Visits/Hospitalizations Among Patients With Type 1 Diabetes eFigure 4. Crude Rates of Hypoglycemia-Related ED Visits/Hospitalizations Among Patients With Type 2 Diabetes eReferences. [file jamanetwopen-3-e1919099-s001.pdf]

Supplementary Online Content

McCoy RG, Lipska KJ, Van Houten HK, Shah ND. Association of cumulative multimorbidity, glycemic control, and medication use with hypoglycemia-related emergency department visits and hospitalizations among adults with diabetes. *JAMA Netw Open*. 2020;3(1):e1919099. doi:10.1001/jamanetworkopen.2019.19099

eTable 1. ICD-9 and ICD-10 Codes Used to Ascertain Study Conditions
eTable 2. Adjusted Risk Factors for Hypoglycemia-Related ED Visits/Hospitalizations—Type 1 vs Type 2 Diabetes
eTable 3. Differential Effect of Multimorbidity as a Function of Age
eTable 4. Adjusted Risk Factors for Hypoglycemia-Related ED Visits/Hospitalizations—Secondary Analysis Examining Individual Comorbidities in the Overall Population
eTable 5. Adjusted Risk Factors for Hypoglycemia-Related ED Visits/Hospitalizations—Secondary Analysis Examining Individual Comorbidities Among Patients With Type 1 and Type 2 Diabetes
eFigure 1. Study Design
eFigure 2. Study Population
eFigure 3. Crude Rates of Hypoglycemia-Related ED Visits/Hospitalizations Among Patients With Type 1 Diabetes
eFigure 4. Crude Rates of Hypoglycemia-Related ED Visits/Hospitalizations Among Patients With Type 2 Diabetes
eReferences.

This supplementary material has been provided by the authors to give readers additional information about their work.

© 2020 McCoy RG et al. *JAMA Network Open*.

eTable 1. ICD-9 and ICD-10 Codes Used to Ascertain Study Conditions.

Health condition	ICD-9 codes	ICD-10 codes
Hypoglycemia	251.0, 251.1, 251.2, 270.3, 962.3 or 250.8x (and not 259.8, 272.7, 681.xx, 682.xx, 686.9x, 707.1-707.9, 709.3, 730.0-730.2, 731.8)	E08.641, E08.649, E09.641, E09.649, E10.641, E10.649, E11.641, E11.649, E13.641, E13.649, E15, E16.0, E16.1, E16.2, T38.3X1A, T38.3X1D, T38.3X1S, T38.3X2A, T38.3X2D, T38.3X2S, T38.3X3A, T38.3X3D, T38.3X3S, T38.3X4A, T383X4D, T38.3X4S, T38.3X5A, T383X5D, T38.3X5S
	ICD-9 codes from Ginde, et al[1]; ICD-10 codes were adapted by study investigators based on clinical expertise	
Dementia	046.1x, 290.1x, 290.2x, 290.3, 290.4x, 291.2, 292.82, 294.1x, 294.2x, 331.0, 331.1x, 331.2, 331.6, 331.82, 331.89	A81.0x, F01.5x, F02.8x, F03.9x, F10.27, F10.97, F13.27, F13.97, F18.97, F19.17, F19.27, F19.97, G30.x, G31.0x, G31.1, G31.83, G31.85
	Adapted from cohort definition in NQF-endorsed quality measure "Antipsychotic Use in Persons with Dementia" and American Academy of Neurology's "Diagnosis (active) dementia group" value set (found in VSAC)	
End-stage renal disease	585.5, 585.6, 403.01, 403.11, 403.91, 404.02, 404.03, 404.12, 404.13, 404.92, 404.93, V45.11	N18.5, N18.6, I12.0, I13.11, I13.2
	Investigator developed algorithm based on clinical expertise and literature review	
Chronic kidney disease, stage 3-4	585.3, 585.4	N18.3, N18.4
	Investigator developed algorithm based on clinical expertise and literature review	
Myocardial infarction	410.xx, 412	I21.xx, I22.x, I25.2
	Adapted from AHRQ CCS category 100 [2]	
Heart failure	398.91, 402.01, 402.11, 402.91, 404.01, 404.03, 404.11, 404.13, 404.91, 404.93, 428.xx	I09.81, I11.0, I13.0, I13.2, I50.xx
	Adapted from the cohort for the NQF-endorsed CMS "Hospital 30-day, all-cause, risk-standardized readmission rate (RSRR) following heart failure (HF) hospitalization" measure (ACO-37; NQF #0330)[3 4]	
Cerebrovascular disease	430, 431, 432.x, 433.xx, 434.xx, 435.x, 436, 437.x, 438.xx, V12.54	G45.0, G45.1, G45.2, G45.8, G45.9, G46.x, I60.xx, I61.x (except I61.0), I62.xx, I63.xxx, I65.xx, I66.xx, I67.8x (except I67.83, I67.84), I67.9, I69.xxx, Z86.73
	Adapted from "Cerebrovascular disease, Stroke, TIA" value set from Quality Insights of Pennsylvania (QIP) from VSAC	
Chronic obstructive pulmonary disease	490, 491.xx, 492.x, 494.x, 496	J40, J41.x, J42, J43.x, J44.x, J47.x
	Definition taken from the chronic obstructive pulmonary disease and asthma chronic disease group in the NQF-endorsed CMS "All-Cause Unplanned Admissions for Patients with Multiple Chronic Conditions" measure (ACO-38)[5]	
Cancer (except non-melanoma skin cancer)	14x.xx, 15x.xx, 160.x-165.x, 170.x-172.x, 174.x-176.x, 179-189.x, 19x.xx, 200.xx-208.xx (except 203.x1, 204.x1, 205.x1, 206.x1, 207.x1, 208.x1)	C00.x-C14.x, C15.x-C26.x, C3x.xx, C40.xx-C41.x, C43.x, C4A.xx, C45.x-C49.xx, C50.xxx, C51.x-C58, C60.x-C63.x, C64.x-C68.x, C69.xx-C72.x, C73-C75.x, C76.x-C80.x, C7A.xx, C81.xx-C96.x (except C90.x1, C91.x1, C92.x1, C93.x1, C94.x1, C95.x1)
	Taken from AHRQ CCS categories 11-22; 24-43 [2]	
Cirrhosis	571.2, 571.5, 571.6	K70.3x, K74.3, K74.4, K74.5, K74.6x
	Taken from "Cirrhosis" value set from MITRE (found on VSAC)	

© 2020 McCoy RG et al. *JAMA Network Open*.

Proliferative retinopathy	361.xx, 362.02, 362.53, 369.xx, 379.23	H33.xxx, E11.359x, H35.359, H54.xx, H43.1x
	Adapted from Glasheen, et al[6] and the NQF-endorsed CMS "Risk-Standardized Acute Admission Rates for Patients with Diabetes" measure (ACO-36) [7]	
Peripheral neuropathy	250.6x, 354.x, 355.x, 356.9, 357.2, 713.5	E10.40, E10.42, E11.40, E11.42, E13.42, G56.x, G57.x, G58.x, G60.9, M14.6x
	Adapted from Glasheen, et al[6] and the NQF-endorsed CMS "Risk-Standardized Acute Admission Rates for Patients with Diabetes" measure (ACO-36) [7]	
Hypertension	401.x, 402.xx, 403.xx, 404.xx, 405.xx	I10, I11.x, I12.x, I13.xx, I15.x, I16.x
	Adapted from "Hypertension" value sets from MITRE and Quality Insights of Pennsylvania (found on VSAC)	
Arthritis	711.2x, 714.xx (except 714.81), 715.xx, 720.x, 725	M05.xxx, M06.xxx, M12.0xx, M35.2, M45.x, M46.1, M46.8x, M46.9x, M49.8x, M35.3
	Taken from AHRQ CCS categories 202-203, 205 [2]	
Urinary incontinence	625.6, 788.30, 788.31, 788.32, 788.33, 788.34, 788.38, 788.39, 788.91	N39.3, N39.41, N39.42, N39.46, N39.490, N39.492, N39.498, R32, R39.81
	Investigator developed algorithm based on clinical expertise and literature review	
Depression	311, 290.13, 290.21, 290.43, 296.2x, 296.3x, 296.82, 298.0, 301.12, 309.0, 309.1, 309.28	F01.51, F32.xx (except F32.81), F33.xx, F34.8x, F43.21, F43.23
	Adapted from "Depression" value set from Quality Insights of Pennsylvania (found on VSAC)	
Falls	E88x.x, E987.x	W00.xxxx, W01.xxxx, W03.xxxx, W04.xxxx, W05.xxxx, W06.xxxx, W07.xxxx, W08.xxxx, W09.xxxx, W10.xxxx, W11.xxxx, W12.xxxx, W13.xxxx, W14.xxxx, W15.xxxx, W16.xxxx, W17.xxxx, W18.xxxx, W19.xxxx, Y30.XXXA
	Investigator developed algorithm based on clinical expertise and literature review	

Abbreviations: ACO, Accountable Care Organization; CCS, Clinical Classification Software ; MI, myocardial infarction; VSAC, United States National Library of Medicine Value Set Authority Center

Analytic Note: All code sets were reviewed for relevance and validity by study investigators experienced in diabetes management and diabetes-related health care delivery research. In circumstances where no validated code sets existed (specifically: falls, urinary incontinence), relevant diagnosis codes were identified by study investigators based on clinical experience and review of pertinent literature.

The Ginde ICD-9 algorithm has been used extensively to identify hypoglycemia-related utilization events, including hospitalizations and emergency department visits, despite its original development in the ED setting.[1] It is used by the Centers for Disease Control and Prevention (CDC) to ascertain hospitalizations and ED visits for hypoglycemia (https://gis.cdc.gov/grasp/diabetes/DiabetesAtlas.html#) using data from the National Inpatient Sample (NIS) and the National Emergency Department Sample (NEDS), respectively.

All ICD9 and ICD10 codes for diabetes complications of retinopathy and neuropathy were adapted from the validated Diabetes Complications Severity Index (DCSI)[6] and also the National Quality Forum (NQF)-endorsed CMS "Risk-Standardized Acute Admission Rates for Patients with Diabetes" measure (ACO-36)[7]. For retinopathy, the DCSI and ACO-36 definitions include both proliferative and non-proliferative retinopathy codes, and proliferative retinopathy codes were extracted by the study investigators. Rationale for focusing on proliferative rather than all retinopathy is that American Diabetes Association (ADA) and Department of Veterans Affairs/ Department of Defense (VA/DoD) specify that patients with severe non-proliferative diabetic retinopathy (NPDR) and proliferative diabetic retinopathy (PDR) should be treated to less stringent glycemic targets.

Because we relied on diagnosis codes to identify patients with PDR, we sought to capture the likely ways that PDR is billed by providers, which means looking not only for codes associated with PDR but also the ways that PDR

© 2020 McCoy RG et al. *JAMA Network Open*.

presents in any given patient. Specifically, PDR (ICD9 362.02) is defined by the presence of neovascularization arising from the disc or retinal vessels, which can lead to pre- retinal and vitreous hemorrhage (ICD9 379.23), fibrosis, and traction retinal detachment (ICD9 361.xx).[8] We included cystoid macular degeneration (ICD9 362.53) because clinically significant macular edema can lead to vision loss. We also included blindness and low vision (ICD9 369.x) because this reflects end-stage or clinically significant eye disease. We did not include diabetic ophthalmologic disease (ICD9 250.5x); background diabetic retinopathy (ICD9 362.01); other background retinopathy and retinal vascular changes (ICD9 362.1x); or retinal hemorrhage, retinal exudates and deposits, and retinal edema (ICD9 362.81-362.83) because these diagnoses are not necessarily reflective of severe NPDR or PDR.

© 2020 McCoy RG et al. *JAMA Network Open*.

eTable 2. Adjusted Risk Factors for Hypoglycemia-Related ED Visits/ Hospitalizations—Type 1 vs Type 2 Diabetes. These Poisson regression analyses examine the associations between patient characteristics and potential hypoglycemia risk factors with hypoglycemia-related ED visits and hospitalizations among adults with type 1 and type 2 diabetes, with all factors adjusted for simultaneously. The total number of chronic conditions was calculated from among dementia, end-stage renal disease, stage 3-4 chronic kidney disease, myocardial infarction, heart failure, cerebrovascular disease (transient ischemic attack/stroke), chronic obstructive pulmonary disease, cancer (except for non-melanoma skin cancer), cirrhosis, proliferative retinopathy, peripheral neuropathy, hypertension, arthritis, urinary incontinence, depression, and falls. Prior history of a hypoglycemia-related ED visit/hospitalization ("prior hypoglycemia") was considered separately and therefore was not included in the total count of guideline-specified chronic conditions. *With or without other glucose-lowering medications (not insulin, sulfonylurea).

	**Type 1 Diabetes**		**Type 2 Diabetes**	
	IRR (95% CI)	P	IRR (95% CI)	P
**Age group**				
18-44 years	Ref	Ref	Ref	Ref
45-64 years	1.37 (0.91, 2.06)	0.13	0.86 (0.63, 1.17)	0.33
65-74 years	0.87 (0.54, 1.41)	0.57	1.30 (0.96, 1.76)	0.09
≥75 years	1.10 (0.66, 1.84)	0.72	1.64 (1.21, 2.24)	0.002
**Gender**				
Male	Ref	Ref	Ref	Ref
Female	1.07 (0.83, 1.39)	0.60	0.92 (0.83, 1.02)	0.12
**Race/ethnicity**				
White	Ref	Ref	Ref	Ref
Black	1.07 (0.76, 1.52)	0.68	1.35 (1.20, 1.52)	<0.001
Hispanic	0.46 (0.26, 0.8)	0.01	0.83 (0.71, 0.98)	0.02
Asian	2.14 (1.05, 4.4)	0.04	1.00 (0.76, 1.32)	0.98
Unknown	1.04 (0.52, 2.06)	0.91	0.99 (0.74, 1.31)	0.97
**Annual household income**				
<$40,000	Ref	Ref	Ref	Ref
$40,000-$49,999	1.51 (0.95, 2.40)	0.08	0.98 (0.83, 1.16)	0.81
$50,000-$59,999	0.95 (0.54, 1.67)	0.86	0.96 (0.80, 1.15)	0.63
$60,000-$74,999	1.26 (0.79, 2.01)	0.33	0.87 (0.73, 1.03)	0.11
$75,000-$99,999	0.93 (0.58, 1.49)	0.77	0.82 (0.68, 0.98)	0.03
≥$100,000	0.78 (0.51, 1.19)	0.25	0.60 (0.50, 0.72)	<0.001
Unknown	1.36 (0.89, 2.06)	0.15	0.97 (0.79, 1.20)	0.75
**Prior severe hypoglycemia-related ED visits/hospitalizations**	9.79 (7.23, 13.26)	<0.001	5.78 (4.96, 6.74)	<0.001
**Comorbidities**				
0-1	Ref	Ref	Ref	Ref

© 2020 McCoy RG et al. *JAMA Network Open.*

2	2.06 (1.38, 3.09)	<0.001	1.10 (1.35, 1.91)	<0.001
3	1.76 (1.12, 2.76)	0.01	2.22 (1.87, 2.64)	<0.001
4	3.43 (2.22, 5.32)	<0.001	2.47 (2.05, 2.97)	<0.001
5	2.74 (1.63, 4.63)	<0.001	3.00 (2.46, 3.67)	<0.001
6	5.32 (3.18, 8.92)	<0.001	3.21 (2.53, 4.07)	<0.001
7	1.61 (0.62, 4.17)	0.33	3.57 (2.68, 4.75)	<0.001
≥8	1.94 (0.67, 5.58)	0.22	4.50 (3.31, 6.12)	<0.001
**Index HbA_{1c} level**				
≤5.6%	1.13 (0.38, 3.41)	0.82	1.48 (1.14, 1.93)	0.003
5.7 - 6.4%	1.33 (0.68, 2.58)	0.41	1.08 (0.91, 1.29)	0.38
6.5 - 6.9%	Ref	Ref	Ref	Ref
7.0 - 7.9%	1.72 (1.01, 2.92)	0.05	0.99 (0.84, 1.16)	0.87
8.0 - 8.9%	1.86 (1.08, 3.20)	0.02	1.14 (0.95, 1.36)	0.16
9.0 - 9.9%	1.50 (0.81, 2.75)	0.20	1.15 (0.94, 1.42)	0.18
≥10.0%	2.17 (1.23, 3.83)	0.01	1.14 (0.93, 1.40)	0.20
**Treatment regimen**				
Other medications			Ref	Ref
Sulfonylurea*			6.78 (4.95, 9.27)	<0.001
Basal insulin*	Ref	Ref	12.62 (8.94, 17.82)	<0.001
Basal insulin + sulfonylurea*	0.88 (0.23, 3.43)	0.86	14.16 (10.02, 20.00)	<0.001
Bolus insulin*	1.11 (0.48, 2.56)	0.80	23.66 (14.87, 37.66)	<0.001
Bolus insulin + sulfonylurea*	-	--	19.12 (10.63, 34.37)	<0.001
Basal + bolus insulins*	1.46 (0.68, 3.14)	0.33	28.68 (21.04, 39.10)	<0.001
Basal + bolus insulins + sulfonylurea*	0.50 (0.10, 2.43)	0.39	27.50 (19.75, 38.30)	<0.001
No fills			4.09 (2.94, 5.69)	<0.001

Abbreviations: HbA_{1c}, hemoglobin A_{1c}.

© 2020 McCoy RG et al. *JAMA Network Open*.

eTable 3. Differential Effect of Multimorbidity as a Function of Age. We examined the possible interaction between patient age and increasing count of comorbidities on the risk of experiencing hypoglycemia requiring ED visits and hospitalizations by calculating the adjusted incidence rate ratios (IRRs) for hypoglycemia-related ED visits/hospitalizations separately for each age group. Comorbidity count was analyzed as a continuous variable. Poisson regression models were adjusted for all remaining factors, including gender, race/ethnicity, annual household income, diabetes type, prior severe hypoglycemia-related ED/hospital care, index hemoglobin A_{1c}, and the glucose-lowering treatment regimen.

	**Overall**		**Type 1 Diabetes**		**Type 2 Diabetes**	
	IRR (95% CI)	P	IRR (95% CI)	P	IRR (95% CI)	P
**18-44 years**	1.55 (1.37, 1.77)	<0.001	1.60 (1.27, 2.03)	<0.001	1.55 (1.32, 1.82)	<0.001
**45-64 years**	1.28 (1.22, 1.34)	<0.001	1.31 (1.20, 1.43)	<0.001	1.27 (1.20, 1.35)	<0.001
**65-74 years**	1.25 (1.20, 1.30)	<0.001	1.15 (0.98, 1.33)	0.08	1.26 (1.21, 1.31)	<0.001
**≥75 years**	1.08 (1.03, 1.13)	0.001	0.87 (0.73, 1.03)	0.10	1.10 (1.05, 1.15)	<0.001

© 2020 McCoy RG et al. *JAMA Network Open*.

eTable 4. Adjusted Risk Factors for Hypoglycemia-Related ED Visits and Hospitalizations—Secondary Analysis Examining Individual Comorbidities in the Overall Population. In this Poisson regression analysis, the association between each comorbid health condition, rather than the total count of comorbidities, and hypoglycemia-related ED visits and hospitalizations was assessed. All variables were adjusted for simultaneously, allowing for the examination of the independent associations between each of these risk factors and the risk of hypoglycemia-related ED visits/hospitalizations. *With or without other glucose-lowering medications (not insulin, sulfonylurea).

	IRR (95% CI)	p-value
**Age group**		
18-44 years	Ref	Ref
45-64 years	0.97 (0.76, 1.24)	0.79
65-74 years	1.30 (1.02, 1.66)	0.04
≥75 years	1.70 (1.32, 2.19)	<0.001
**Gender**		
Male	Ref	Ref
Female	0.94 (0.86, 1.04)	0.25
**Race/ethnicity**		
White	Ref	Ref
Black	1.31 (1.17, 1.47)	<0.001
Hispanic	0.78 (0.67, 0.91)	0.001
Asian	1.05 (0.81, 1.36)	0.71
Unknown	1.00 (0.77, 1.29)	0.99
**Household income**		
<$40,000	Ref	Ref
$40,000-$49,999	1.01 (0.87, 1.18)	0.88
$50,000-$59,999	0.95 (0.80, 1.12)	0.53
$60,000-$74,999	0.90 (0.76, 1.12)	0.19
$75,000-$99,999	0.82 (0.69, 0.97)	0.02
≥$100,000	0.62 (0.52, 0.73)	<0.001
Unknown	0.99 (0.83, 1.19)	0.96
**Diabetes type**		
Type I	1.35 (1.16, 1.57)	<0.001
Type II	Ref	Ref
**Prior severe hypoglycemia-related ED visits/hospitalizations**	6.29 (5.49, 7.21)	<0.001
**Comorbidities**		
None	0.63 (0.44, 0.89)	0.01
Dementia	1.02 (0.84, 1.23)	0.88
ESRD	1.43 (1.18, 1.73)	<0.001
CKD, stages 3-4	1.37 (1.22, 1.53)	<0.001

© 2020 McCoy RG et al. *JAMA Network Open*.

Myocardial infarction	1.41 (1.20, 1.65)	<0.001
Heart failure	1.06 (0.94, 1.2)	0.32
Stroke/TIA	1.20 (1.07, 1.35)	0.002
COPD	1.15 (1.02, 1.29)	0.02
Cancer (except non-melanoma skin cancer)	0.93 (0.80, 1.08)	0.34
Cirrhosis	1.18 (0.85, 1.65)	0.31
Proliferative retinopathy	1.15 (0.96, 1.39)	0.13
Peripheral neuropathy	1.27 (1.15, 1.4)	<0.001
Hypertension	1.10 (0.90, 1.34)	0.36
Arthritis	1.07 (0.96, 1.19)	0.23
Urinary incontinence	1.06 (0.87, 1.29)	0.54
Depression	1.31 (1.16, 1.49)	<0.001
Falls	1.44 (1.22, 1.70)	<0.001
**Index HbA_{1c} levels**		
≤5.6%	1.44 (1.11, 1.85)	0.01
5.7 - 6.4%	1.09 (0.92, 1.30)	0.32
6.5 - 6.9%	Ref	Ref
7.0 - 7.9%	1.04 (0.89, 1.22)	0.61
8.0 - 8.9%	1.19 (1.01, 1.41)	0.04
9.0 - 9.9%	1.16 (0.95, 1.41)	0.15
≥10.0%	1.22 (1.01, 1.47)	0.04
**Glucose-lowering treatment regimen**		
Other medications	Ref	Ref
Sulfonylurea*	6.69 (4.90, 9.16)	<0.001
Basal insulin*	12.48 (8.86, 15.58)	<0.001
Basal insulin + sulfonylurea*	13.83 (9.81, 19.50)	<0.001
Bolus insulin*	23.72 (16.06, 35.06)	<0.001
Bolus insulin + sulfonylurea*	19.26 (10.71, 34.61)	<0.001
Basal + bolus insulins*	27.62 (20.28, 37.62)	<0.001
Basal + bolus insulins + sulfonylurea*	26.17 (18.81, 36.39)	<0.001
No fills	4.24 (3.05, 5.90)	<0.001

Abbreviations: HbA_{1c}, hemoglobin A_{1c}. CKD, chronic kidney disease stages 3-4; COPD, chronic obstructive pulmonary disease; ESRD, end-stage renal disease; TIA, transient ischemic attack.

© 2020 McCoy RG et al. *JAMA Network Open*.

eTable 5. Adjusted Risk Factors for Hypoglycemia-Related ED Visits/ Hospitalizations—Secondary Analysis Examining Individual Comorbidities Among Patients With Type 1 and Type 2 Diabetes. In this Poisson regression analysis, the association between each comorbid health condition, rather than the total count of comorbidities, and hypoglycemia-related ED visits/hospitalizations was assessed separately for patients with type 1 and type 2 diabetes. All variables were adjusted for simultaneously, allowing for the examination of the independent contribution of each of these risk factors to the risk of hypoglycemia-related ED visits/hospitalizations. *With or without other glucose-lowering medications (not insulin, sulfonylurea).

	**Type 1 Diabetes**		**Type 2 Diabetes**	
	IRR (95% CI)	P	IRR (95% CI)	P
**Age group**				
18-44 years	Ref	Ref	Ref	Ref
45-64 years	1.34 (0.88, 2.04)	0.18	0.88 (0.64, 1.19)	0.40
65-74 years	0.99 (0.60, 1.63)	0.97	1.34 (0.99, 1.82)	0.06
≥75 years	1.43 (0.83, 2.48)	0.20	1.73 (1.27, 2.36)	0.001
**Gender**				
Male	Ref	Ref	Ref	Ref
Female	0.98 (0.75, 1.28)	0.86	0.94 (0.85, 1.04)	0.23
**Race/ethnicity**				
White	Ref	Ref	Ref	Ref
Black	1.11 (0.78, 1.57)	0.57	1.36 (1.20, 1.53)	<0.001
Hispanic	0.51 (0.29, 0.90)	0.02	0.82 (0.70, 0.96)	0.02
Asian	2.02 (0.99, 4.14)	0.06	0.99 (0.75, 1.30)	0.94
Unknown	1.14 (0.57, 2.27)	0.71	1.00 (0.76, 1.31)	0.98
**Annual household income**				
<$40,000	Ref	Ref	Ref	Ref
$40,000-$49,999	1.37 (0.86, 2.17)	0.18	0.97 (0.82, 1.15)	0.74
$50,000-$59,999	0.81 (0.46, 1.43)	0.47	0.96 (0.80, 1.15)	0.65
$60,000-$74,999	1.27 (0.79, 2.04)	0.32	0.86 (0.72, 1.03)	0.09
$75,000-$99,999	0.92 (0.57, 1.47)	0.72	0.82 (0.68, 0.98)	0.03
≥$100,000	0.77 (0.50, 1.18)	0.22	0.59 (0.49, 0.72)	<0.001
Unknown	1.27 (0.82, 1.96)	0.28	0.96 (0.78, 1.18)	0.68
**Prior severe hypoglycemia-related ED visits/hospitalizations**	8.56 (6.26, 11.70)	<0.001	5.54 (4.75, 6.47)	<0.001
**Comorbidities**				
None	1.00 (0.54, 1.84)	1.00	0.49 (0.31, 0.78)	0.002
Dementia	0.75 (0.44, 1.30)	0.31	1.13 (0.92, 1.39)	0.24
ESRD	0.91 (0.45, 1.82)	0.78	1.52 (1.24, 1.86)	<0.001
CKD, stages 3-4	1.24 (0.86, 1.78)	0.25	1.38 (1.23, 1.56)	<0.001
Myocardial infarction	2.43 (1.64, 3.62)	<0.001	1.26 (1.06, 1.50)	0.01

© 2020 McCoy RG et al. *JAMA Network Open.*

Heart failure	0.68 (0.44, 1.03)	0.07	1.15 (1.01, 1.31)	0.04
Stroke/TIA	1.15 (0.81, 1.65)	0.43	1.23 (1.09, 1.39)	0.001
COPD	0.63 (0.42, 0.96)	0.03	1.23 (1.09, 1.38)	0.001
Cancer (except non-melanoma skin cancer)	0.60 (0.33, 1.08)	0.09	0.95 (0.81, 1.12)	0.54
Cirrhosis	0.75 (0.23, 2.43)	0.63	1.28 (0.91, 1.81)	0.15
Proliferative retinopathy	1.02 (0.67, 1.56)	0.92	1.13 (0.92, 1.39)	0.24
Peripheral neuropathy	1.59 (1.20, 2.11)	0.001	1.21 (1.09, 1.35)	<0.001
Hypertension	1.45 (0.91, 2.30)	0.12	1.03 (0.82, 1.28)	0.82
Arthritis	1.31 (0.96, 1.79)	0.09	1.04 (0.93, 1.16)	0.52
Urinary incontinence	1.36 (0.77, 2.42)	0.29	1.04 (0.84, 1.28)	0.74
Depression	1.52 (1.11, 2.07)	0.01	1.27 (1.11, 1.46)	0.001
Falls	2.00 (1.33, 3.02)	0.001	1.33 (1.11, 1.60)	0.002
**Index HbA_{1c} levels**				
≤5.6%	1.38 (0.46, 4.17)	0.56	1.45 (1.11, 1.88)	0.01
5.7 - 6.4%	1.42 (0.73, 2.78)	0.30	1.07 (0.89, 1.27)	0.48
6.5 - 6.9%	Ref	Ref	Ref	Ref
7.0 - 7.9%	1.52 (0.89, 2.61)	0.12	0.98 (0.83, 1.16)	0.84
8.0 - 8.9%	1.75 (1.02, 3.02)	0.04	1.13 (0.95, 1.35)	0.17
9.0 - 9.9%	1.38 (0.75, 2.54)	0.30	1.16 (0.94, 1.42)	0.18
≥10.0%	2.03 (1.15, 3.60)	0.02	1.14 (0.92, 1.39)	0.23
**Glucose-lowering treatment regimen**				
Other medications	--	--	Ref	Ref
Sulfonylurea*	--	--	6.70 (4.89, 9.16)	<0.001
Basal insulin*	1	Ref	12.39 (8.77, 17.51)	<0.001
Basal insulin + sulfonylurea*	0.78 (0.20, 3.04)	0.72	13.95 (9.87, 19.71)	<0.001
Bolus insulin*	1.08 (0.47, 2.48)	0.87	24.02 (15.09, 38.22)	<0.001
Bolus insulin + sulfonylurea*	--	--	19.46 (10.82, 35.01)	<0.001
Basal + bolus insulins*	1.46 (0.68, 3.14)	0.34	28.20 (20.66, 38.49)	<0.001
Basal + bolus insulins + sulfonylurea*	0.46 (0.09, 2.24)	0.34	27.38 (19.65, 38.15)	<0.001
No fills			4.13 (2.97, 5.74)	<0.001

Abbreviations: HbA_{1c}, hemoglobin A_{1c}.

© 2020 McCoy RG et al. *JAMA Network Open.*

eFigure 1. Study Design. Schematic demonstrating the time periods during which study outcomes and the independent (predictor) variables were assessed. The index date was set to the last available hemoglobin A_{1c} result in 2015.

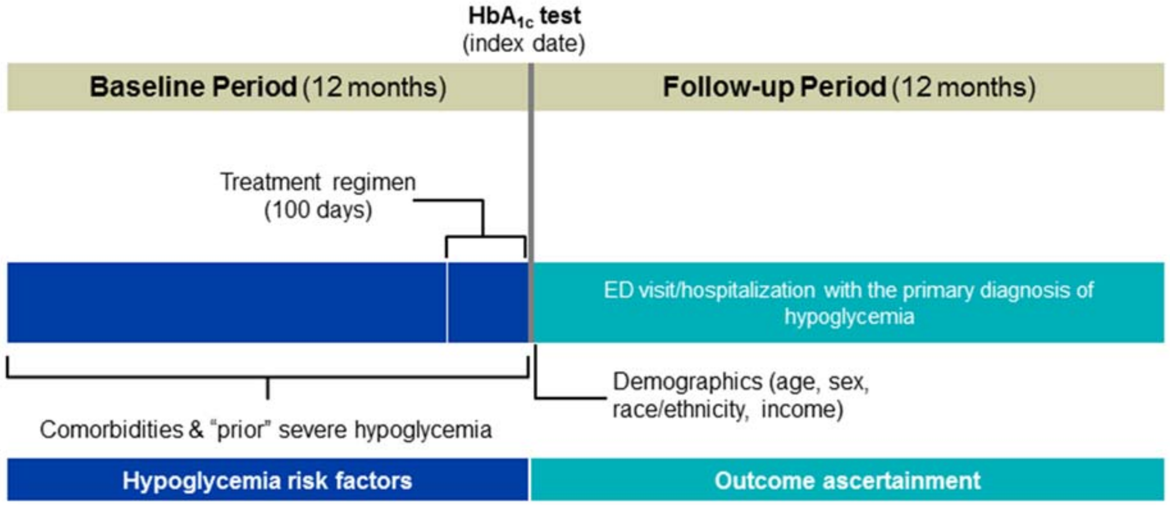

© 2020 McCoy RG et al. *JAMA Network Open*.

eFigure 2. Study Population.

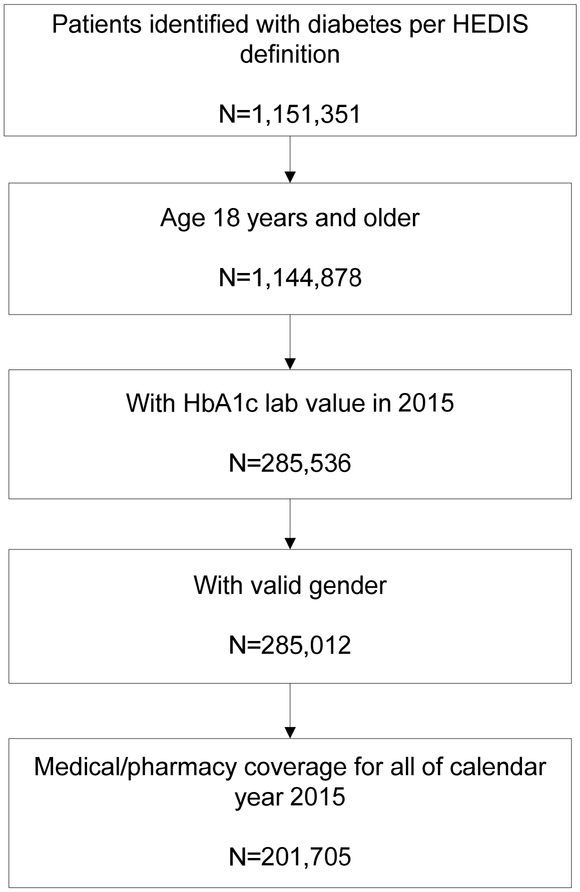

© 2020 McCoy RG et al. *JAMA Network Open*.

eFigure 3. Crude Rates of Hypoglycemia-Related ED Visits/Hospitalizations Among Patients With Type 1 Diabetes. Crude (unadjusted) rates of hypoglycemia-related ED visits/hospitalizations experienced by adults with type 1 diabetes, stratified by **(A)** patient age; **(B)** annual household income in $U.S.; **(C)** total number of comorbid health conditions from among those specified by the American Diabetes Association (ADA) and the U.S. Department of Veterans Affairs/ Department of Defense (VA/DoD) diabetes management guidelines; (D) hemoglobin A_{1c}; and (E) the individual comorbidities specified by the ADA and the VA/DoD diabetes management guidelines. Rates of hypoglycemia-related ED visits/hospitalizations were calculated as the total number of hospitalizations and emergency department visits with the primary/first diagnosis of hypoglycemia per 1000 persons with the characteristic of interest (for example, age ≥75 years) per one year.

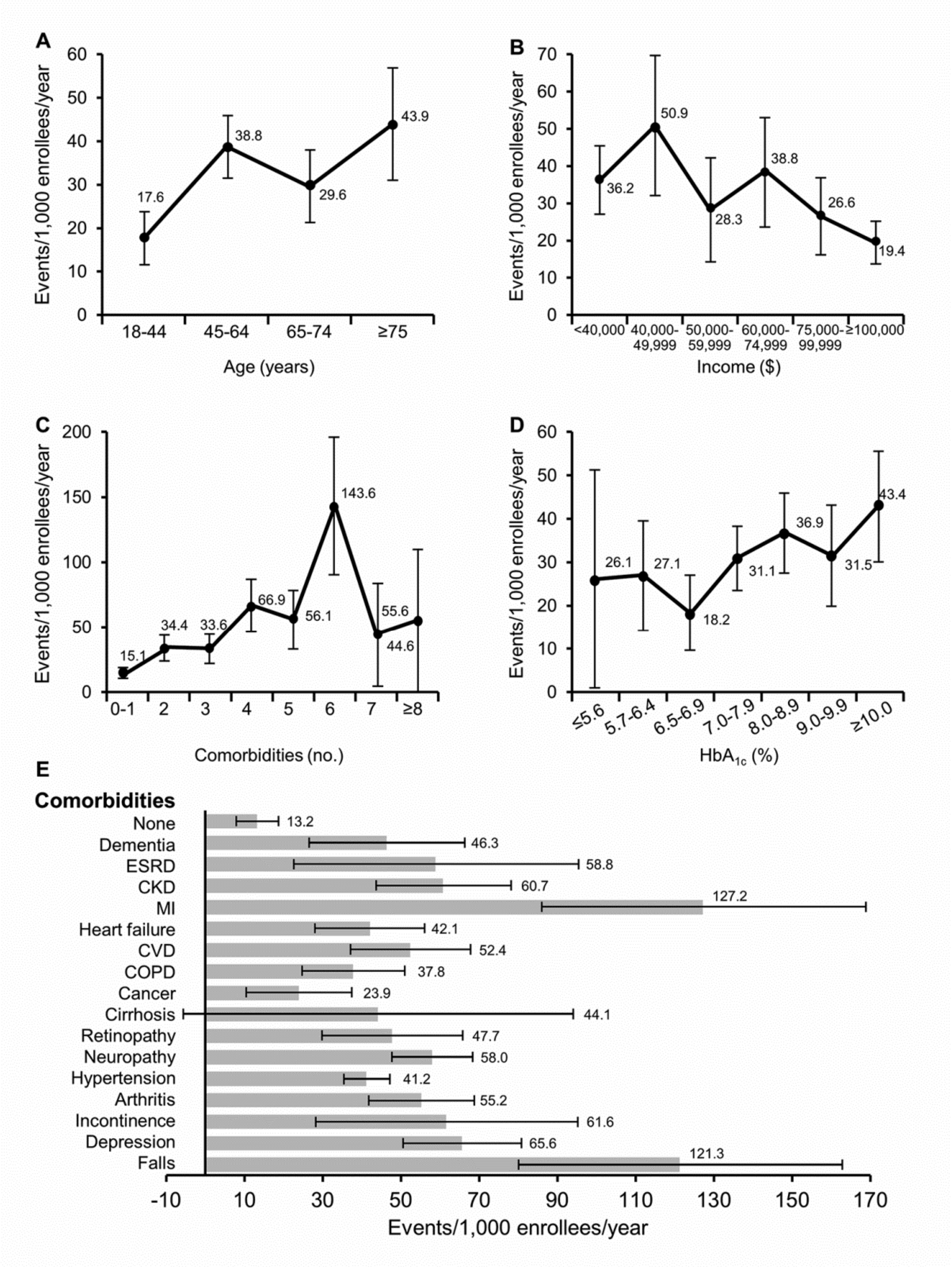

© 2020 McCoy RG et al. *JAMA Network Open*.

eFigure 4. Crude Rates of Hypoglycemia-Related ED Visits/Hospitalizations Among Patients With Type 2 Diabetes. Crude (unadjusted) rates of hypoglycemia-related ED visits/hospitalizations experienced by adults with type 2 diabetes, stratified by (A) patient age; (B) annual household income in $U.S.; (C) total number of comorbid health conditions from among those specified by the American Diabetes Association (ADA) and the U.S. Department of Veterans Affairs/ Department of Defense (VA/DoD) diabetes management guidelines; (D) hemoglobin A1c; (E) the individual comorbidities specified by the ADA and the VA/DoD diabetes management guidelines; and (F) the glucose-lowering treatment regimen. Rates of hypoglycemia-related ED visits/hospitalizations were calculated as the total number of hospitalizations and emergency department visits with the primary/first diagnosis of hypoglycemia per 1000 persons with the characteristic of interest (for example, age ≥75 years) per one year.

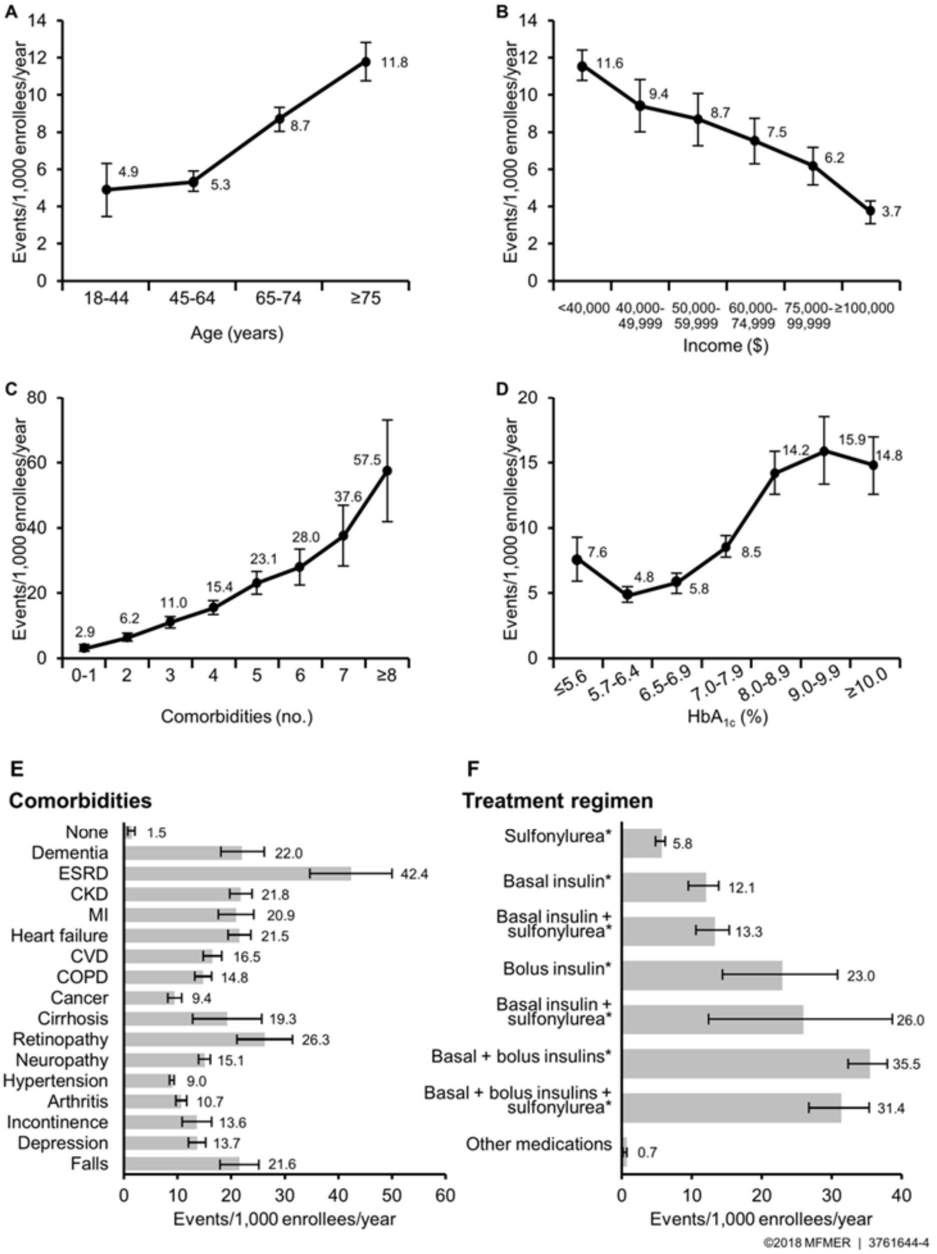

© 2020 McCoy RG et al. *JAMA Network Open*.

eReferences.

1. Ginde AA, Blanc PG, Lieberman RM, et al. Validation of ICD-9-CM coding algorithm for improved identification of hypoglycemia visits. *BMC Endocr Disord* 2008;8:4. doi: 1472-6823-8-4 [pii] 10.1186/1472-6823-8-4 [published Online First: 2008/04/03]
2. Elixhauser A, Steiner C, Palmer L. Clinical Classifications Software (CCS), 2015 Rockville, MD: United States Agency for Healthcare Research and Quality; 2016 [updated March 2016; cited 2018 September 5]. Available from: http://www.hcup-us.ahrq.gov/toolssoftware/ccs/ccs.jsp accessed September 5 2018.
3. NQF. NQF #0330 Centers for Medicare & Medicaid Services (CMS) "Hospital 30-day, all-cause, risk-standardized readmission rate (RSRR) following heart failure (HF) hospitalization" measure: Centers for Medicare & Medicaid Services (CMS); 2018 [updated July 12, 2018; cited 2018 September 7]. Available from: http://www.qualityforum.org/ accessed September 7 2018.
4. CMS. Accountable Care Organization (ACO) #37 Risk-Standardized Acute Admission Rates for Patients With Heart Failure. Version 2.0: Centers for Medicare & Medicaid Services; 2015 [updated December 31, 2015; cited 2018 September 7]. Version 2.0:[Available from: https://www.cms.gov/Medicare/Medicare-Fee-for-Service-Payment/sharedsavingsprogram/Downloads/ACO-37.pdf accessed September 7 2018.
5. CMS. Accountable Care Organization (ACO) #38 Risk-Standardized Acute Admission Rates for Patients With Multiple Chronic Conditions. Version 2.0: Centers for Medicare & Medicaid Services; 2015 [updated December 31, 2015; cited 2018 September 7]. Available from: https://www.cms.gov/Medicare/Medicare-Fee-for-Service-Payment/sharedsavingsprogram/Downloads/ACO-38.pdf accessed September 7 2018.
6. Glasheen WP, Renda A, Dong Y. Diabetes Complications Severity Index (DCSI)-Update and ICD-10 translation. *J Diabetes Complications* 2017;31(6):1007-13. doi: 10.1016/j.jdiacomp.2017.02.018 [published Online First: 2017/04/19]
7. CMS. Accountable Care Organization (ACO) #36 Risk-Standardized Acute Admission Rates for Patients With Diabetes: Centers for Medicare & Medicaid Services (CMS); 2015 [updated December 31, 2015; cited 2018 September 5]. Version 2.0, effective 12/31/2015:[Available from: https://www.cms.gov/Medicare/Medicare-Fee-for-Service-Payment/sharedsavingsprogram/Downloads/ACO-36.pdf accessed September 5 2018.
8. Aiello LM. Perspectives on diabetic retinopathy. *Am J Ophthalmol* 2003;136(1):122-35. [published Online First: 2003/07/02]

© 2020 McCoy RG et al. *JAMA Network Open*.
